# Advances in AI-assisted quantification of dry eye indicators

**DOI:** 10.3389/fmed.2025.1628311

**Published:** 2025-07-18

**Authors:** Lingyan Wu, Yuntao Huang, Tao Lv, Chun Xiao, Ying Wang, Shanping Zhao

**Affiliations:** ^1^Hangzhou Lin’an Traditional Chinese Medicine Hospital, Affiliated Hospital of Hangzhou City University, Hangzhou, China; ^2^National Clinical Research Center for Ocular Diseases, Eye Hospital of Wenzhou Medical University, Wenzhou, China; ^3^The First People’s Hospital of Aksu District in Xinjiang, Aksu City, China

**Keywords:** narrative review, ophthalmology, treatment, diagnosis, machine learning, artificial intelligence, dry eye disease

## Abstract

Dry eye disease (DED) is a multifactorial ocular surface disorder characterized by ocular discomfort, visual disturbances, and potential structural damage. The heterogeneous etiology and symptomatology of DED pose significant challenges for accurate diagnosis and effective treatment. In recent years, artificial intelligence (AI), particularly deep learning (DL), has shown substantial promise in improving the objectivity and efficiency of DED assessment. This review provides a comprehensive synthesis of AI-assisted techniques for the quantification of key DED biomarkers, including tear film stability [e.g., tear meniscus height (TMH) and tear film break-up time (TBUT)], meibomian gland morphology, and corneal epithelial damage. We discuss how these technologies enhance diagnostic accuracy, standardize evaluation, and support personalized treatment. Collectively, these advancements underscore the transformative potential of AI in reshaping DED diagnostics and management.

## 1 Introduction

Dry eye disease (DED) is a multifactorial and increasingly prevalent ocular surface disorder characterized by insufficient tear production, excessive tear evaporation, or tear film instability (1). Epidemiological studies estimate that DED affects between 5 and 50% of the global population, with variations attributed to differences in diagnostic criteria and study population (2). While age is a well-established risk factor, modern environmental and behavioral factors—including prolonged screen exposure, air pollution, and widespread use of air conditioning—have contributed to increasing incidence rates among younger individuals (3).

DED presents with a wide range of symptoms, including ocular dryness, foreign body sensation, stinging or burning, photophobia, and blurred vision (2). These symptoms can impair daily functioning, reduce work productivity, and significantly diminish quality of life (2). Moreover, the chronic discomfort associated with DED can lead to psychological comorbidities such as anxiety and depression, further exacerbating the disease burden (4, 5). Despite its clinical significance, DED remains challenging to diagnose and monitor due to the heterogeneity of its presentation and the subjectivity of traditional diagnostic tools. Current evaluations rely heavily on subjective assessments—such as tear break-up time (TBUT), Schirmer’s test, ocular surface staining, and meibography—often depend on clinician expertise and patient cooperation, leading to poor reproducibility and limited diagnostic accuracy (6, 7). Additionally, weak correlations between clinical signs and patient-reported symptoms hinder objective assessment of disease severity and treatment efficacy.

In response to these challenges, artificial intelligence (AI) has emerged as a transformative tool in ophthalmology (8). Advances in machine learning (ML) and deep learning (DL) have enabled an automated analysis of ocular imaging and clinical data, facilitating more objective and reproducible assessments (9, 10). Initially applied in retinal disease detection (11), AI technologies are currently increasingly adapted for anterior segment conditions. In the objective assessment of DED, AI technology can assist doctors in automating the identification and quantification of ocular surface abnormalities and reducing reliance on manual operation and subjective evaluations, thereby improving diagnostic efficiency (12–16) (Table 1). This review synthesizes current advancements in AI-assisted quantification of dry eye indicators and discusses their clinical implications, limitations, and future directions.

**Table 1:** Summary of artificial intelligence models across different indicators.

Type	Quantitative indicators	Study	Data type	Sample size (*N*)	Methods	Key findings	Performance	References
TF	TFBUT	Detection of DED via estimating tear film breakup time (TFBUT)	Smart eye camera (video recordable slit lamp device)	22,172 frames from 158 eyes	DL-central neural network (CNN)	The model displays high accuracy and AUROC in estimating tear film breakup time (TFBUT) using ocular surface videos	For estimating TFBUT: Acc = 78.9% AUROC = 0.877 F1 score = 0.74. For diagnosing DED using ADES criteria: Sens = 77.8% Spec = 85.7% AUROC = 0.813	Shimizu et al. (14)
		Detection of TFBUT	Tear film images	9,089 image patches from 350 eyes	DL–CNN-ResNet50	The model can detect TFBUT with high accuracy using tear film images taken by the non-invasive device	For classifying tear breakup or non-breakup group: Acc = 92.4% Sens = 83.4% Spec = 95.2%	Kikukawa et al. (22)
		Prediction of unstable tear film from clinical data	Multimodal clinical data	432 patients	ML—AdaBoostM1, LogitBoost, RF	The applied ML algorithms outperform the baseline classification scheme (i.e., ZeroR)	Average recall and precision > 0.74	Fineide et al. (24)
	Incomplete blink frames	Detection of DED via blink analysis	Blink videos (collected via keratograph 5 M)	1,019 image sets	DL	The model can analyze blink videos with high accuracy and sensitivity. Incomplete blinking frequency was found to be closely associated with DED symptoms	For 30 FPS videos: balanced accuracy = 95.82% Sens = 99.38% IoU = 0.8868 Dice = 0.9251	Zheng et al. (25)
	Tear film break-up area (TFBA)	Detection of DED	Ocular surface videos	244 eyes	Deep transfer learning	Deep transfer learning model displays high accuracy in detecting DED from ocular surface video data. Lower paracentral cornea was identified as the most important region by the CNN model for the detection of DED	For discriminating DED and normal eyes: AUROC = 0.98	Abdelmotaal et al. (23)
		Detection of fluorescent tear film break-up area	Slit-lamp data	50 subjects	DL-CNN	The model achieves robust performance with good agreement with standard methods to measure tear film stability (i.e., TFBUT)	*R* = 0.9 between CNN-BUT and TFBUT test. As a metric, CNN-BUT is statistically significantly lower in patients with DED (*p* < 0.05). At a given cutoff of 5 s Sens = 83% Spec = 95% AUROC = 0.96	Su et al. (15)
PEE	PEE	Grading punctate epithelial erosion (PEE)	Anterior slit-lamp images	1,046 images	Deep NN	The model can grade PEE with good accuracy, illustrating its potential utility as a training platform	Segmentation performance: IoU = 0.937 Grading performance: Acc = 76.5% AUROC = 0.94	Qu et al. (13)
TM	TMH	Measurement of tear meniscus height	Oculus camera photographs	510 images	DL—CNN	The model demonstrates robust performance in segmenting, identifying, and quantifying the tear meniscus	For corneal segmentation task: Dice = 0.99 IoU = 0.98 For tear meniscus detection: Dice = 0.92 IoU = 0.86	Wang et al. (16, 18, 39)
		Segmentation of lower tear meniscus images	OCT	6,658 images	DL	The proposed approach displays robust segmentation and localization of the lower tear meniscus	Acc > 99.2% Sens > 96.3% Spec > 99.8% IoU > 0.931	Stegmann et al. (19)
		Measurement of tear meniscus height	Smartphone images	1,021 images	DL	The model demonstrates robust performance in automated tear meniscus height measurement for potential DED diagnosis	Dice coefficient = 0.9868; Acc = 95.39%	Nejat et al. (20)
		Measurement of tear meniscus height	Keratograph 5M	3,894 images	ALNN	To propose an automatic measurement method for TMH based on convolutional neural networks to handle diverse datasets	Color image modality: average MIoU of 0.9578; infrared image modality: average MIoU of 0.9290.	Wang et al. (21)
MG	MG density	Segment and diagnose MGD via meibomian gland density	Infrared meibography	4,006 meibography images	DL and TL	The model illustrates the utility of using meibomian density in improving the accuracy of meibography analysis	Segmentation performance: Acc = 92% and repeatability = 100% MG density in total eyelids’ performance: Sens = 88%	Zhang et al. (31)
	Regions of MG atrophy	Segment and quantify MG atrophy	Meibography images	706 images	DL	The proposed DL can segment the total eyelid and meibomian gland atrophy regions with high accuracy and consistency. The model also achieves accurate Meiboscore grading accuracy, outperforming human clinical teams	Meiboscore grading: Acc = 95.6%. For eyelid segmentation: Acc = 97.6%; IoU = 95.5%. For atrophy segmentation: Acc = 95.4%; IoU = 66.7%; RMSD for atrophy prediction = 6.7%	Wang et al. (34)
		Measure MG atrophy	Meibography images	497 images	DL—NPID, using the CNN backbone	The model can automatically analyze MG atrophy and categorize gland characteristics via hierarchical clustering with good performance, outperforming a human clinician in Meiboscore grading accuracy	Meiboscore grading accuracy with pretrained model = 80.9% Meiboscore grading accuracy without pretrained model = 63.6%	Yeh et al. (37)
	Ghost glands	Quantification of MG morphology	Infrared meibography images	1,443 images	DL	The model can automatically segment meibomian glands, identify ghost glands, and quantitatively analyze gland morphological features with good performance	Segmentation performance: IoU = 0.63 Identification of ghost glands: Sens = 84.4% Spec = 71.7%	Wang et al. (35)
	MG	Development of an automated DL method to segment MG	Infrared meibography	728 images	DL	The model demonstrates robust performance in segmenting MG	Segmentation performance: precision = 83% recall = 81% F1 score = 84% dice = 0.84 AUROC = 0.96	Setu et al. (32)
		Quantification of MG irregularities	Meibography images	90 images	ML—conditional generative adversarial network (cGAN)	The proposed technique outperforms state-of-the-art methods for the detection and analysis of the dropout area of MGD, as well as provides a notable improvement in quantifying the irregularities of infrared MG images	F1 = 0.825 average Pompeiu–Hausdorff distance = 0.664 Mean loss area = 30.1% *R* = 0.962 and 0.968 between automatic and manual analyses	Khan et al. (36)
	MG, eyelid	Segment MG and eyelids, analyze the MG area, and estimate the Meiboscore	Meibography images	1,600 images	DL—ResNet	The DL model demonstrates robust automated performance in the evaluation of MG morphology, ranging from segmentation to Meiboscore, comparable to human ophthalmologists	Meiboscore classification performance: Acc = 73.01% on the validation set	Saha et al. (33)
Others	Eyelid margin signs	Identification of lid margin signs for DED	Anterior segment images	832 images	DL	The model can identify lid margin signs with high sensitivity and specificity	For posterior lid margin: AUROC = 0.979. For lid margin irregularity: AUROC = 0.977. For lid margin vascularization: AUROC = 0.98. For meibomian gland orifice (MGO) retroplacement: AUROC = 0.963. For MGO plugging: AUROC = 0.968. For posterior lid margin: Sens = 97.4% Spec = 93.8%.	Wang et al. (16, 18, 39)
	DED probability score, ASOCT	Evaluation of a DL approach to diagnose DED using AS-OCT images	AS-OCT	27,180 images from 151 eyes	DL—VGG19	The model displays robust performance in detecting DED, especially compared to the standard clinical DED test and similar to cornea specialists	Acc = 84.62% Sens = 86.36% Spec = 82.35%	Chase et al. (38)
	Corneal epithelial	Detection of DED	OCT epithelial mapping	228 eyes	ML—RF and LR	Inclusion of OCT corneal epithelial mapping can facilitate the diagnosis of DED with high sensitivity and specificity	Sens = 86.4% Spec = 91.7% AUROC = 0.87	Edorh et al. (40)

## 2 AI-driven quantification of DED biomarkers

### 2.1 Tear meniscus height (TMH)

**Figure 1:**
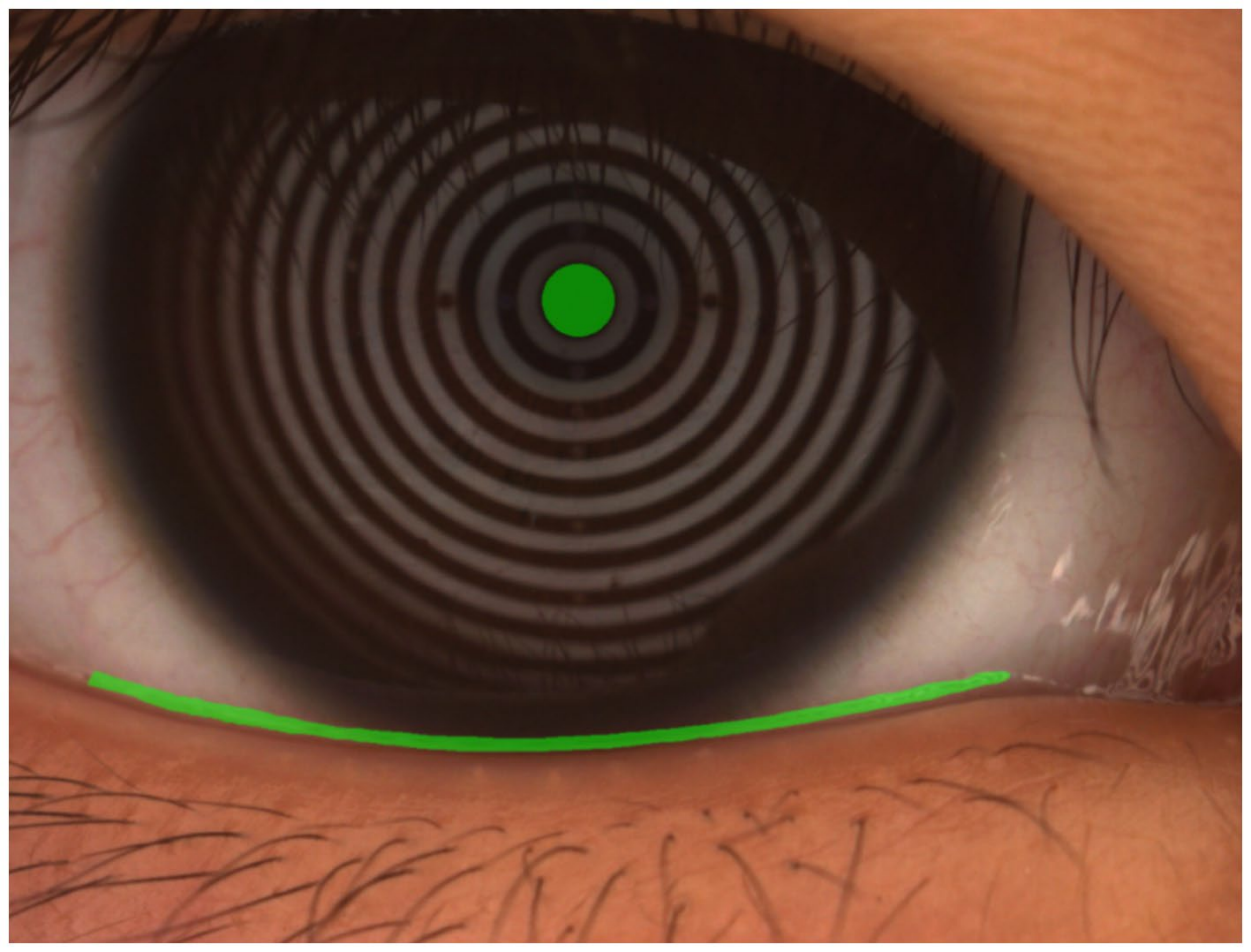
Manual measurement of tear meniscus height.

The tear meniscus reflects the dynamic balance between tear production and drainage and can be used to quantify tear volume by measuring the height of the tear film at the lower eyelid margin (17). Traditionally, this measurement is performed manually under slit-lamp microscopy, a method subject to considerable operator-dependent variability and inefficiency (Figure 1). AI-based approaches have significantly improved the reliability and scalability of TMH assessment. Automated deep learning models enable high-precision TMH quantification with minimal human intervention, ensuring greater diagnostic consistency. For example, Wang et al. (18) applied enhanced U-Net architectures to TMH segmentation and corneal boundary detection in images captured using oculus-captured images, achieving a dice similarity coefficient (DSC) of 0.92 and an intersection-over-union (IoU) of 0.86 for TMH detection, along with near-perfect corneal segmentation [dice similarity coefficient (DSC): 0.99, IoU: 0.98]. Similarly, Stegmann et al. (19) developed a DL-based segmentation technique for optical coherence tomography (OCT) images, reporting sensitivity above 96%, specificity over 99%, and Jaccard indices exceeding 93% across two segmentation strategies.

In an effort to enhance accessibility, researchers have explored mobile-compatible solutions. Nejat et al. (20) introduced a DL algorithm for TMH measurement using smartphone-captured images, analyzing features such as iris diameter, eyelid position, and pupillary light reflex to achieve a dice coefficient of 98.68% and an overall accuracy of 95.39%. This development highlights the feasibility of AI-assisted, point-of-care DED screening in low-resource settings.

However, generalizability remains a concern in existing models, many of which rely on a single image modality and lack validation on external datasets. To address this, Wang et al. (21) proposed a two-stage DL framework that incorporates dual-modality data from a Keratograph 5M (K5M) system. Their model achieved a mean intersection-over-union (MIoU) of 0.9578 for color images and 0.9290 for infrared images, demonstrating robust performance across imaging conditions.

### 2.2 Tear film break-up time (TBUT)

Tear film break-up time (TBUT) is a fundamental indicator of tear film stability and is widely used in the clinical diagnosis of dry eye disease. Based on the measurement technique, TBUT can be classified into fluorescein TBUT (FBUT) and non-invasive TBUT (NIBUT). In FBUT, fluorescein dye is instilled into the tear film, and the time from a complete blink to the first appearance of a dry spot is recorded. A TBUT of less than 10 s typically indicates tear film instability, which is a key feature of DED.

Although TBUT testing is simple and widely used, it is inherently subjective and sensitive to multiple variables, including examiner skill, lighting conditions, and patient cooperation. These limitations hinder reproducibility and restrict its use in standardized, large-scale screening. AI-based systems offer a promising solution by enabling automated TBUT measurement through video and image analysis. Deep learning algorithms can detect tear film break-up points, analyze blink dynamics, and predict instability using multimodal clinical data. These tools minimize observer bias, improve diagnostic consistency, and facilitate high-resolution, continuous monitoring of tear film behavior.

#### 2.2.1 Video-based automated tear film break-up detection

Early efforts to apply AI in TBUT analysis demonstrated high diagnostic performance. Su et al. developed a DL model capable of automatically identifying tear film break-up regions in slit-lamp video recordings from 80 participants, achieving an area under the receiver operating characteristic curve (AUROC) of 0.96, with 83% sensitivity and 95% specificity for DED detection (15). Expanding on this, Shimizu et al. (14) developed an AI algorithm using portable slit-lamp videos to measure TBUT, reporting an AUC of 0.813, a sensitivity of 77.8%, and a specificity of 85.7%. Kikukawa et al. (22) further optimized this approach by using a KOWA DR-1α device combined with convolutional neural networks (CNNs), yielding an AUC of 0.898, a sensitivity of 77.8%, and a specificity of 85.7%. More recently, Abdelmotaal et al. (23) utilized single-frame image analysis, where their CNN model achieved an AUC of 0.98 in identifying DED using single-frame images. Notably, their model identified the lower paracentral cornea as the most informative region for classification, as revealed through network activation maps.

#### 2.2.2 Multimodal data-driven prediction of tear film stability

Beyond image-based evaluation, multimodal data integration has further enhanced TBUT prediction. Fineide et al. analyzed clinical datasets incorporating variables such as Schirmer test results, ocular surface staining (OSS), meibomian gland secretion quality (MQ), tear osmolarity, blink rate, and Ocular Surface Disease Index (OSDI) scores (24). Their model effectively predicted unstable tear film with average recall and precision exceeding 0.74 (24). Zheng et al. (25) proposed a novel approach that combined blink video analysis, symptom questionnaires, and ocular surface assessment data (via Keratograph 5 M) to train the DL system for real-time detection of incomplete blinking. Their system achieved outstanding performance with a balanced accuracy of 95.82%, a sensitivity of 99.38%, an IoU of 0.8868, and a dice coefficient of 0.9251 in 30 Frames Per Second videos, offering a high-precision tool for dynamic behavioral analysis. However, most current studies remain single-center and lack external validation. Broader, multicenter investigations are needed to confirm model generalizability and clinical utility across diverse populations and imaging devices.

### 2.3 Corneal fluorescein staining (CFS)

Corneal staining is a fundamental technique for evaluating epithelial damage on the ocular surface in patients with dry eye disease. Dyes such as fluorescein and lissamine green are commonly applied to identify devitalized or damaged epithelial cells, with fluorescein producing bright green fluorescence that highlights areas of disruption (26).

Traditional grading systems, including the Norn, Oxford, and National Eye Institute (NEI) scales, rely on clinician-dependent visual interpretation of staining patterns. These systems assess the density, area, and distribution of punctate epithelial erosions (PEEs). However, their subjective nature introduces significant variability, influenced by factors such as examiner experience and ambient lighting conditions. This lack of standardization often limits inter-observer consistency and diagnostic reliability.

Recent advances in AI and computer vision have addressed these limitations by enabling automated, pixel-level quantification of corneal staining. Deep learning algorithms can currently segment corneal regions, identify stained areas, and compute objective metrics such as lesion area, staining density, and topographic distribution. These methods enhance diagnostic reproducibility and reduce reliance on manual grading. For instance, Qu et al. (13) developed a deep neural network (DNN) framework that automatically segments corneal regions, extracts image patches, and grades PEEs. Their model achieved an IoU of 0.937 for corneal segmentation and an AUC of 0.940 for detecting punctate staining, thereby outperforming conventional grading reproducibility. Despite these promising developments, a major challenge remains: the coexistence of multiple grading schemes with differing criteria. The lack of a universally accepted standard hinders the development of unified AI models capable of cross-system compatibility. Continued research is needed to harmonize classification protocols and validate AI-based grading systems across diverse datasets and clinical workflows. Deng et al. (27) built a new Fine-grained Knowledge Distillation Corneal Staining Score (FKD-CSS) with a dual-decoder architecture to simultaneously detect kerato-corneal lesions and generate continuous scores from coarse-annotated data. Compared to ResNext and DenseNet, it achieves higher consistency and accuracy (*r* = 0.898, AUC = 0.881). However, widespread clinical adoption faces substantial barriers. Algorithm performance is highly dependent on consistent, high-quality image capture. Factors such as fluorescein concentration, instillation-to-imaging time, lighting, patient cooperation (blink artifacts), and camera settings introduce significant variability, challenging algorithmic robustness.

### 2.4 Meibomian gland morphology

**Figure 2:**
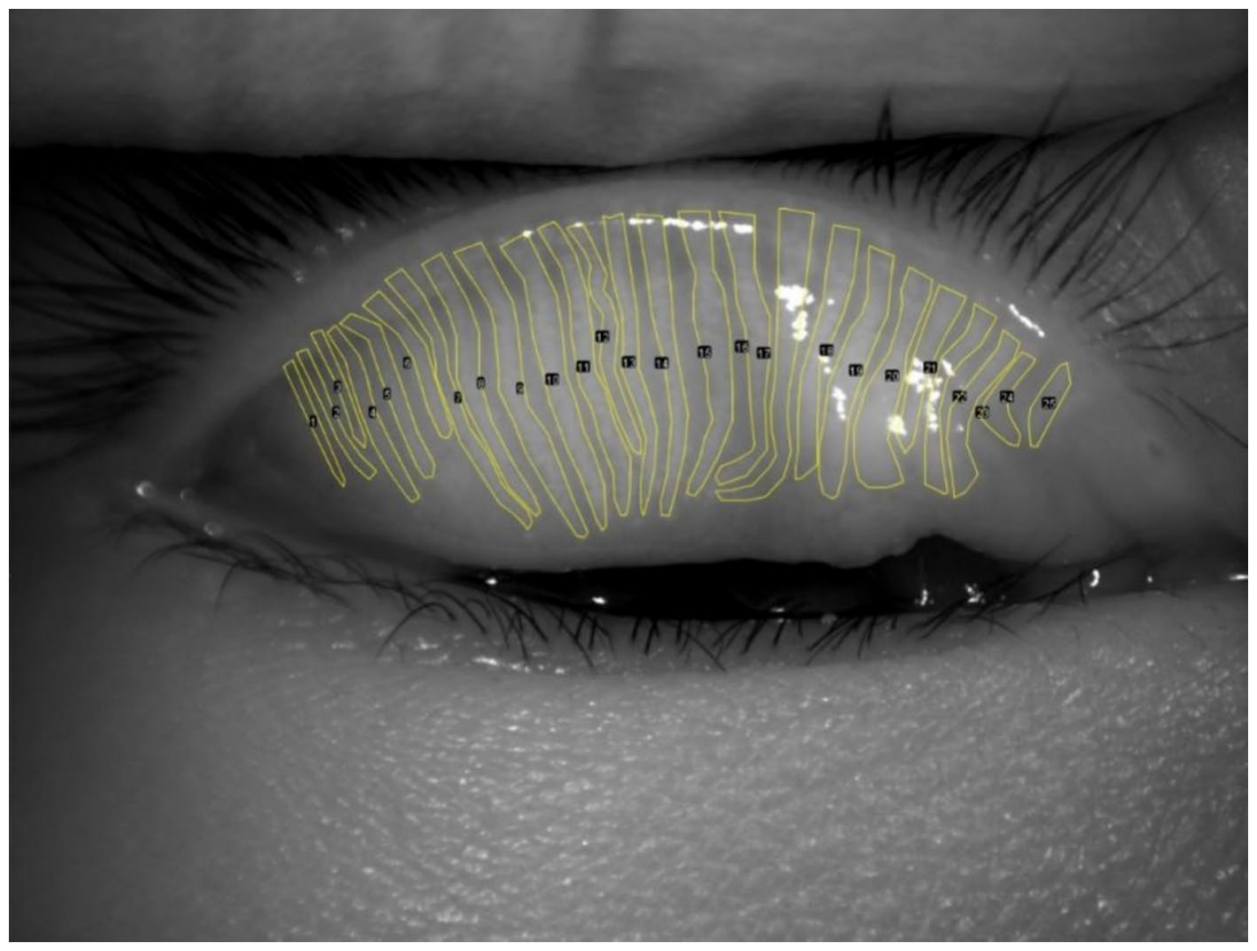
Manual segmentation of the meibomian gland.

Advanced imaging methods offer precise and detailed views of the meibomian glands, aiding in the diagnosis of evaporative DED caused by meibomian gland dysfunction (MGD) (28). MGD is a leading cause of evaporative DED, with gland morphology and atrophy severity directly linked to tear film instability (29). Traditional assessments rely on subjective interpretation of meibography images, often using semi-quantitative grading systems that are time-consuming and susceptible to inter-observer variability (Figure 2). Recent developments in AI have enabled automated, high-precision methods for evaluating MG morphology, including segmentation, dropout quantification, and atrophy assessment (30). DL and generative adversarial network (GAN)-based models have demonstrated near-human performance in analyzing meibography images, enabling fast, scalable, and reproducible evaluations. Moreover, unsupervised learning frameworks are emerging to facilitate severity grading without the need for manually annotated datasets.

Zhang et al. (31) developed a transfer learning-based system for MG segmentation and density assessment, achieving 92% segmentation accuracy, 88% sensitivity, and 81% specificity for MGD diagnosis, with a processing speed of 100 ms per image. Setu et al. (32) utilized a U-Net model to segment MG in meibography images, reporting 83% segmentation accuracy and automated quantification of gland length, width, and curvature within 1.33 s per image. Saha et al. (33) introduced a DL model to quantify MG area, proportion, and Meiboscore (a standardized atrophy grading system), achieving 73.01% classification accuracy on validation datasets.

Wang et al. (34) proposed a DL framework for segmenting atrophic MG regions, achieving 97.6% eyelid segmentation accuracy, 95.4% gland segmentation accuracy, and a root mean square deviation (RMSD) of 6.7% in atrophy percentage prediction. Their subsequent study on “ghost gland” detection (degenerated MG lacking visible structure) achieved 84.4% sensitivity and 71.7% specificity using a hybrid segmentation–classification model (35). Khan et al. (36) applied conditional generative adversarial networks (CGANs) to infrared meibography, achieving an F1 score of 0.825 and near-perfect correlation (*r* = 0.968) with manual analysis for atrophy quantification. To address the scarcity of annotated data, Yeh et al. (37) pioneered an unsupervised feature learning method to classify MG atrophy severity without annotated training data. Using hierarchical clustering, their model achieved 80.9% accuracy in Meiboscore grading, enabling phenotype-driven quantitative assessment. Despite these achievements, several challenges persist. The acquisition of high-quality, expert-labeled meibography datasets remains resource-intensive. Additionally, the majority of current frameworks do not account for demographic and clinical covariates—such as age, sex, and ethnicity—which may influence MG morphology. Future research should integrate these variables to better understand population-specific risk factors for MGD and enhance the generalizability of AI-driven assessments.

## 3 Emerging AI applications in multimodal and novel biomarker analysis

Beyond traditional biomarkers, AI is expanding the diagnostic landscape of DED by incorporating advanced imaging modalities and integrating multimodal data sources. Techniques such as AS-OCT, lid margin imaging, and corneal epithelial thickness mapping are currently being combined with functional parameters (e.g., blink rate, osmolarity, and OSDI scores). These integrated approaches offer a more holistic evaluation of ocular surface health and DED subtypes. Chase et al. (38) pioneered a DL model to diagnose DED using AS-OCT images, generating a probability score based on corneal and conjunctival structural features. The model demonstrated an accuracy rate of 84.62%, a sensitivity of 86.36%, and a specificity of 82.35% outperforming traditional tests such as Schirmer’s test and corneal staining, while matching the diagnostic efficacy of TBUT and OSDI, highlighting AS-OCT’s potential as a standalone imaging-based diagnostic tool (38). Wang et al. (39) used ML to analyze slit-lamp images for lid margin abnormalities—a critical yet underutilized biomarker in DED. Their model achieved exceptional AUCs for detecting posterior lid margin features: 0.979 (circular patterns), 0.977 (irregularities), and 0.980 (vascularization), demonstrating AI’s ability to extract subtle, clinician-overlooked signs with high precision (39). However, the study was limited by a relatively small dataset and low prevalence of positive cases (e.g., posterior lid margin rounding), which may bias model outputs and reduce generalizability. Edorh et al. (40) developed an AI-assisted scoring system integrating OCT-derived corneal epithelial thickness maps, which quantifies spatial epithelial heterogeneity to optimize DED diagnosis. The model achieved a sensitivity of 86.4% and a specificity of 91.7%, offering a novel objective framework that complements functional tear film assessments (40). Despite these promising results, OCT’s limited ability to distinguish between the precorneal tear film and the corneal epithelium introduces potential uncertainty in interpretation.

Collectively, these innovations highlight the growing role of AI in identifying novel DED biomarkers and enabling multimodal diagnostic strategies. By moving beyond conventional tear film metrics, AI-driven systems are poised to redefine clinical paradigms in dry eye diagnosis and personalized management.

## 4 Future prospects

The integration of AI into DED diagnostics and management holds transformative potential, yet several critical pathways must be prioritized to bridge current advancements with real-world clinical utility. First, CNNs dominate perceptual vision tasks, LLMs redefine language capabilities, and traditional ML remains the pragmatic powerhouse for structured data analysis, especially under constraints. The optimal choice emerges from a careful evaluation of the specific application requirements against the inherent strengths and limitations of each paradigm. Future studies lie not only in advancing each paradigm but also in intelligently integrating them (e.g., using CNN/LLM features for traditional models, using traditional models to verify LLM outputs) to harness their combined strengths. Second, the “black-box” nature of complex models such as deep CNNs and LLMs remains a significant barrier to clinical adoption. Future studies must prioritize developing domain-specific interpretability techniques that provide clinically meaningful explanations for AI-driven diagnoses (e.g., highlighting lesion characteristics in images and identifying key phrases in clinical notes influencing a prediction). Techniques such as attention visualization and concept-based explanations need tailoring to medical contexts. Third, the push toward portable and decentralized solutions—such as smartphone-based TMH monitoring or home-use infrared meibography—will democratize access to early screening, particularly in underserved regions. Lightweight AI models (e.g., quantized neural networks) optimized for edge devices will be essential to support real-time analysis without compromising accuracy. Fourth, addressing data heterogeneity through standardized imaging protocols and federated learning frameworks will enhance model generalizability across diverse populations and devices. Collaborative efforts to establish open-access, annotated datasets encompassing global demographics are urgently needed. Fifth, the ethical and regulatory landscape must evolve to ensure patient trust, emphasizing transparent AI decision-making (e.g., explainable heatmaps for clinicians) and robust data encryption for sensitive ocular biometrics. Finally, the transition from diagnostic AI to therapeutic AI—such as closed-loop systems linking real-time tear film assessments with automated drug delivery or neuromodulation—could revolutionize chronic DED management.

To fully realize this vision, interdisciplinary collaboration among ophthalmologists, data scientists, and regulatory bodies is imperative. Prospective multicenter trials validating AI tools against gold-standard metrics, alongside cost-effectiveness analyses, will accelerate clinical adoption. As AI continues to unravel novel biomarkers and redefine diagnostic thresholds, it promises not only to enhance precision medicine but also to reshape global DED care paradigms from reactive treatment to proactive, patient-centered prevention.

## 5 Conclusion

In conclusion, AI has shown considerable potential in the quantitative evaluation and management of dry eye disease DED. AI-driven technologies, such as machine learning algorithms and advanced imaging techniques, offer more accurate and efficient diagnostic capabilities, which facilitate early disease detection and the development of personalized treatment plans. These innovations are expected to enhance clinical decision-making, reduce the burden on healthcare providers, and improve patient outcomes. However, to fully leverage the advantages of AI in the field of DED, it is essential to address challenges such as data privacy issues, the need for large-scale validation studies, and the integration of AI technologies into clinical workflows.
